# Influence of Different Apical Foramen Morphologies on the Accuracy of Four Electronic Foramen Locators

**DOI:** 10.1055/s-0044-1782214

**Published:** 2024-05-02

**Authors:** Renan D. Furlan, Murilo P. Alcalde, Rodrigo R. Vivan, Michel E. Klymus, Ana G.S. Limoeiro, Marco A.H. Duarte, Bruno C. de Vasconcelos

**Affiliations:** 1 Department of Dentistry, Endodontics and Dental Materials, Bauru Dental School, University of São Paulo, Bauru, SP, Brazil; 2 Post-Graduate Program in Dentistry, School of Pharmacy, Dentistry and Nursing, Federal University of Ceará, Fortaleza, Ceará, Brazil; 3 School of Dentistry of Sobral, Federal University of Ceará, Campus Sobral, Sobral, CE, Brazil

**Keywords:** electronic foramen locator, endodontic, root canal length

## Abstract

**Objective** The aim of this study was to evaluate the accuracy of the Root ZX II (RZX), Raypex 6 (RAY), EPex Pro (EPEX), and CanalPro (CNP) electronic foramen locators (EFLs) in different foraminal morphologies (fully formed foramen, immature foramen with parallel walls, and immature foramen with divergent walls); this article also evaluated the influence of different penetration levels (0.0 mm and −1.0 mm).

**Materials and Methods** Thirty single-rooted human premolars were accessed and had their cervical/middle thirds prepared with SX ProTaper files. The apical foramens (AF) were standardized to 250 µm and the initial root canal length (RCL1) was measured under 16x magnification with aid of a digital caliper. Using the alginate model, electronic measurements (EM) were taken 1.0 mm up to AF (EM1/-1) and at AF (EM1/0), always using adjusted hand K-files. The root apexes were then cross-sectioned 3.0 mm from the foramen; then, new RCL (RCL2) and electronic measurements were performed (EM2/-1 and EM2/0.0). Finally, retropreparations were performed with instruments SX ProTaper files introduced 4.0 mm in the apicocervical direction. Then new RCL (RCL3) and electronic measurements (EM3/-1 and EM3/0) were performed.

**Statistical Analysis** Values were tabulated and tested for normality using the Shapiro–Wilk test, which yielded nonparametric distributions of the data. Data were subjected to the Kruskal–Wallis and Dunn tests to estimate possible differences between devices as a function of foramen morphology and/or apical limit. The significance level was set at 5.0%.

**Results** In general, the EFLs were accurate in determining the RCL. Statistically significant differences were observed between EPEX and RAY at 0.0, when measuring the divergent AF canals ( *p* < 0.05). Regarding the different foramen morphologies in each EFL, RZX and EPEX showed no interference ( *p* > 0.05), whereas RAY and CNP had lower accuracy levels at 0.0 with divergent AF ( *p* < 0.05).

**Conclusion** The four devices evaluated are accurate to determine the RCL in the conditions tested. The apical limit of penetration did not have significant influence on their accuracy. Conversely, the presence of divergence in the AF walls negatively influenced de RAY and CNP precisions at the foraminal level.

## Introduction

The precise location of the apical foramen has a significant impact on the success of endodontic treatment and retreatment. Its definition acts as a guide for professionals to correctly determine the apical limit to clean, shape, and seal the root canal system, thereby minimizing damage to the periapical tissues. 1 2

The role of the electronic foramen locator (EFL) in measuring root canal length (RCL) has been extensively described in the literature. 3 4 5 Nevertheless, certain clinical conditions have been described as detrimental to its accuracy. Factors such as limited apical penetration 6 7 or lack of apical fit 8 and the impossibility of achieving apical patency 9 have been mentioned as possible factors affecting the accuracy of EFLs. These three factors seem to affect the relationship between the apical root canal third and the periapical region.

The mechanism underlying current EFLs depend on the interpretation of impedance, which in turn depends on two other electrical factors: resistance and capacitance. 10 Resistance is associated with the energy delivered by the instrument tip and is closely linked to the apical limit. 10 Capacitance, on the other hand, is related to the energy transmitted along the instrument and is correlated with its adaptation to the root canal walls. 10 It is, therefore, inevitable that clinical conditions that might affect the interpretation of these factors are likely to influence the accuracy of electronic measuring instruments.

Among EFLs, Root ZX II (J. Morita, Tokyo, Japan) and Raypex 6 (VDW GmbH, Munich, Germany) have undergone extensive evaluation, yielding results ranging from unsatisfactory to highly satisfactory, contingent upon clinical conditions. 4 9 11 These devices interpret impedance at two separately radiated frequencies: the first as a function of the quotient of impedance (0.4 and 8 kHz), and the second using the square roots of impedance (0.5 and 8 kHz). Considering the variability in results, new devices have been developed based on these frequency ranges designed to provide accurate and reliable measurements regardless of clinical conditions. Therefore, it is important to evaluate these devices under both ideal and varied conditions.

EPex Pro (MK Life Dental and Medical Products, Porto Alegre, Brazil) and CanalPro (Coltene/Whaledent GmbH, Raiffeisenstrasse, Germany) are two recently launched devices operating on similar mechanisms as the aforementioned devices: EPex Pro like Root ZX II and CanalPro resembling Raypex 6. Although the manufacturers of these devices claim similarities in operating systems with previous devices, they are equipped with different electronic components and have a different design (display size, number of colors on the display, etc.) providing operators with different interpretation parameters. Possible variations related to these differences remain unknown.

To date, no study in the literature has specifically addressed the precision of different EFLs under varying foraminal conditions. These anatomical variations undeniably impact instrument adaptation to canal walls and consequently may affect the accuracy of electronic determinations. Thus, the aim of this study is to determine the accuracy of Root ZX II, Raypex 6, CanalPro, and EPex Pro in different foramen morphologies (fully formed apices, immature foramen with parallel walls, and immature foramen with divergent walls) with variable apical limit penetration depth (0.0 and −1.0 mm). The null hypothesis is that there are no differences between the devices and that the differences in foramen morphology are not significant regardless of apical penetration limit.

## Materials and Methods

### Sample Collection and Preparation

Prior to the study, the sample size was estimated to determine the number of samples required. G*Power for Mac version 3.1 (Heinrich Heine; College of Duesseldorf, Duesseldorf, Germany) was used along with the Wilcoxon-Mann-Whitney test. The data of Vasconcelos et al 9 were considered in this estimation.

After sample size calculation and approval by the local research ethics committee, 30 healthy human mandibular premolars were collected for the study ( *n* = 30). They were straight teeth with a length ranging from 18 to 22 mm and exhibited fully formed apices. After standardized coronal approaches with diamond burs (#1012 and #3081; KG Sorensen, Cotia, Brazil) at high speed, the internal anatomy of the teeth was analyzed. Teeth with two root canals, multiple nonpatent apical foramina (AF), or a diameter exceeding 250 µm were excluded. Cusp tips were also modified to provide flat references for positioning the instruments' penetration stops.

### Cervical Preparation and Instrumentation

Cervical preparation was performed with #17/.08 files (MK Life Dental and Medical Products, Porto Alegre, Brazil) activated by a VDW Silver electric motor (VDW GmbH, Munich, Germany) calibrated to 2N.cm and 800 rpm. Penetration depth was limited to two-thirds of the provisional RCL. Sodium hypochlorite at a concentration of 2.5% (Biodinamica, Ibiporã, Brazil) was used as irrigating solution. The apical foramens were standardized with K-Nitiflex #25 files (Dentsply-Sirona, Ballaigues, Switzerland). A clinical microscope (Alliance, Campinas, Brazil) with 16x magnification was used to determine the baseline RCL. Files were inserted into the canals until their tips were visible in the AF opening. The distance between the rubber stop, aligned with the occlusal reference, and the file tips was measured with a digital caliper (0.001 mm; Mitutoyo, Suzano, Brazil) (RCL1).

### Electronic Measurements

Electrical conductivity was facilitated using an alginate model (Jeltrate II; Dentsply Brasil, Teresópolis, RJ, Brazil). The sample was divided into five subgroups of six samples each for electronic measurements (EM); each subgroup underwent measurements for no more than 30 minutes. Measurements were performed in triplicate by a single operator using matched files at the desired depths. Instruments were used sequentially and alternated for each repetition. Initially, the instrument was inserted to a depth of 1.0 mm just before the AF (−1.0 mm), and file insertion halted upon this depth being indicated on the instrument displays (EM1/-1). Subsequently, the rubber stops were standardized based on occlusal references, files were removed from the root canals, and length was measured with a digital caliper. Measurements were then taken at the level of the foramen (EM1/0.0), indicating when AF was reached (0.0 mm).

For immature tooth tips, 3.0 mm of the apical portion was resected using a Zecrya bur (Dentsply-Sirona) activated at high speed and under abundant irrigation to replicate immature teeth with parallel AF walls. RCL2 was determined as before. EMs were taken 1.0 mm anterior to the AF (EM2/-1) and at the level of the foramen (EM2/0) following the same parameters as for the full apex.

**Fig. 1:**
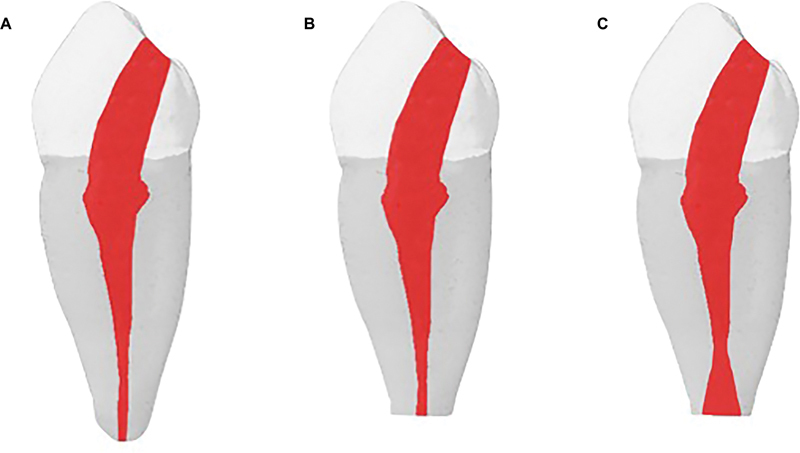
Micro-computed tomography images presenting root canal morphologies: fully formed apices ( **A** ); immature apical foramen with parallel canal walls ( **B** ); and immature apical foramen with divergent canal walls ( **C** ).

Finally, to mimic a tooth with an immature apex and divergent AF walls, a retrograde preparation was performed by inserting #17/.08 instruments 4.0 mm in the apical–cervical direction. RCL3 was determined, and EMs for both apical limits (EM3/-1 and EM3/0) were performed as previously described. Fig. 1 illustrates the tested foramen morphologies.

### Calculation and Statistical Analysis

Mean errors between RCLs and EMs were calculated for two predefined penetration depth limits (0.0 mm and −1.0 mm) using the formulas: Standard error (0.0 mm) = EMx/0-RCLx/0; Standard error (−1.0 mm) = EMx/-1-(RCLx − 1.0 mm).

Statistical analysis involved considering both positive and negative values for measurements beyond and below the AF, respectively. Absolute values of mean errors were utilized. The Shapiro–Wilk test confirmed nonparametric distributions of the data. The Kruskal–Wallis and Dunn tests were applied to determine potential differences between devices based on foramen morphology and/or apical limit. Significance was set at 5.0%.

## Results

Table 1 presents the means, standard deviations, and medians of errors recorded by the EALs across various AF morphologies and apical limits. Comparison between EFLs within each foramen morphology/apical limit revealed a significant difference solely in measurements at the foramen level (0.0 mm) with divergent canal walls. EPex Pro exhibited superior performance, significantly surpassing Raypex 6 ( *p* < 0.05).

**Table 1:** Mean, standard deviation and median of error (mm) provided by each device electronical measurement in different foraminal conditions and penetration levels

Device	Complete apex						Immature apex											
							Parallel apical foramen walls						Expulsive apical foramen walls					
	0.0			−1.0			0.0			−1.0			0.0			−1.0		
	Mean *	SD	Median *	Mean *	SD	Median *	Mean *	SD	Median *	Mean *	SD	Median *	Mean *	SD	Median *	Mean *	SD	Median *
Root ZX II	0.26	0.14	0.25 a A	0.30	0.18	0.27 a A	0.25	0.13	0.28 a A	0.36	0.27	0.30 a A	0.34	0.23	0.31 ab A	0.38	0.33	0.31 a A
Raypex 6	0.27	0.14	0.29 a A	0.35	0.21	0.32 a A	0.29	0.18	0.31 a A	0.41	0.26	0.33 a A	0.49	0.33	0.46 b B	0.49	0.33	0.36 a A
EPex Pro	0.28	0.16	0.26 a A	0.32	0.21	0.30 a A	0.29	0.18	0.29 a A	0.42	0.27	0.38 a A	0.34	0.23	0.30 a A	0.39	0.24	0.41 a A
CanalPro	0.28	0.16	0.27 a A	0.29	0.20	0.27 a A	0.31	0.20	0.32 a AB	0.41	0.31	0.40 a A	0.43	0.25	0.39 ab B	0.49	0.30	0.39 a A

Abbreviation: SD, standard deviation.

* Mean error and median calculated in terms of absolute values of the determinations.

a,b Different lower case letters indicate statistically significant differences according to the Kruskal–Wallis and Dunn tests ( *p* < 0.05), considering the devices in each condition and penetration level.

A,B Different upper case letters indicate statistically significant differences according to the Kruskal–Wallis and Dunn tests ( *p* < 0.05), considering each device at different conditions taking in account each penetration level.

Regarding individual EFL performance across different foramen morphologies, notable differences were observed. Raypex 6 accuracy varied significantly with divergent canal walls compared to other morphologies ( *p* < 0.05). Similarly, CanalPro's accuracy significantly differed between divergent canal walls and complete apices ( *p* < 0.05). However, no significant differences were noted when considering the apical penetration limit for each device ( *p* > 0.05).

Tables 2 and 3 depict the distribution of EFL measurements across the three foramen morphologies. Acceptable measurements were those falling within a tolerance margin of ± 0.5 mm. At the foramen level (0.0 mm), the accuracy of EFLs was notably influenced by foramen morphology. Measurements were less accurate for immature AF with divergent walls (60–80%) compared to complete apices or immature with parallel root canal walls (90–100%). When inserted 1.0 mm below the AF, in general, all EFLs demonstrated reduced accuracy compared to the values obtained at 0.0 mm, except for Root ZX II in complete AF (86.7%). Across other EFLs/morphologies, accuracy remained below 80%, with no significant differences observed in foramen morphologies.

**Table 2:** File tip position relative to the apical foramen for measurements performed to 0.0

Distance from apical foramen (mm)	Root ZX II						Raypex 6						EPex Pro						CanalPro					
	Complete apex		Immature apex				Complete apex		Immature apex				Complete apex		Immature apex				Complete apex		Immature apex			
			Parallel walls		Expulsive walls				Parallel walls		Expulsive walls				Parallel walls		Expulsive walls				Parallel walls		Expulsive walls	
	*n*	%	*n*	%	*n*	%	*n*	%	*n*	%	*n*	%	*n*	%	*n*	%	*n*	%	*n*	%	*n*	%	*n*	%
< −0.51 a	3	10	0	0	6	20	4	13.3	2	6.7	12	40	3	10	3	10	6	20	3	10	2	6.67	10	33.3
−0.50 to −0.01 a	26	86.7	22	73.3	21	70	24	80	19	63.3	17	56.7	24	80	19	63.3	22	73.3	27	90	19	63.3	20	66.7
0.00	0	0	0	0	0	0	0	0	0	0	0	0	0	0	0	0	0	0	0	0	1	3.3	0	0
0.01 to 0.50	1	3.3	8	26.7	3	10	2	6.7	9	30	1	3.3	3	10	8	26.7	2	6.7	0	0	8	26.7	0	0
> 0.51	0	0	0	0	0	0	0	0	0	0	0	0	0	0	0	0	0	0	0	0	0	0	0	0

a Negative value indicates file position short (or coronal) to the apical foramen.

**Table 3:** File tip position during measurements performed short of the apical foramen (−1.0 mm)

Distance from apical foramen (mm)	Root ZX II						Raypex 6						CanalPro						EPex Pro					
	Complete apex		Immature apex				Complete apex		Immature apex				Complete apex		Immature apex				Complete apex		Immature apex			
			Parallel walls		Expulsive walls				Parallel walls		Expulsive walls				Parallel walls		Expulsive walls				Parallel walls		Expulsive walls	
	*n*	%	*n*	%	*n*	%	*n*	%	*n*	%	*n*	%	*n*	%	*n*	%	*n*	%	*n*	%	*n*	%	*n*	%
< −1.51 a	1	3.4	1	3.4	7	23.3	0	0	0	0	8	26.7	0	0	0	0	5	16.7	1	3.34	2	6.7	10	33.3
−1.50 to −1.01 a	10	33.3	7	23.3	16	53.3	13	43.4	7	23.3	11	36.7	8	26.7	3	10	13	43.3	13	43.3	10	33.3	16	53.3
−1.00	0	0	0	0	0	0	0	0	0	0	0	0	0	0	0	0	0	0	0	0	0	0	0	0
−0.50 to −0.01	16	53.3	12	40	6	20	10	33.3	12	40	7	23.3	16	53.3	12	40	9	30	10	33.3	8	26.7	2	6.7
> 0.00	3	10	10	33.3	1	3.4	7	23.3	11	36.7	4	13.3	6	20	15	50	3	10	6	20	10	33.3	2	6.7

a Negative value indicates file position short (or coronal) to the apical foramen.

## Discussion

In this study, the accuracy of four EFLs (Root ZX II, Raypex 6, EPex Pro, and CanalPro) was evaluated during EMs across different foramen morphologies (complete apex, immature foramen with parallel root canal walls, and immature foramen with divergent root canal walls). The impact of the apical limit of penetration on measurements was also assessed. The findings indicate no substantial differences among the evaluated EFLs. However, it is evident that the accuracy of EFLs may diminish in cases of immature AF teeth with divergent root canal walls, which is crucial information for clinicians during procedures. Regarding the apical limit of penetration, it did not significantly influence the accuracy of the tested EFLs. Thus, the null hypothesis was partially rejected.

To achieve the study's objectives, the alginate model was employed, 12 emphasizing the importance of prior cervical preparation 13 and AF standardization. 8 9 The alginate method already has it clinical relevance previously ensured. 14 These procedures aimed to minimize distortions associated with instrument apical adaptation. 8 9 13 Considering the differences in foraminal morphology, apicoectomy was performed to simulate immature teeth with wide AF and parallel root walls. Tapered instruments were utilized to create wide AF with divergent canal walls in extreme conditions.

Regarding the results, all four tested EFLs demonstrated satisfactory accuracy and safety. Irrespective of the apical limit or foramen morphology, the lowest and highest standard errors were 0.26 (Root ZX II, 0.0 mm) and 0.46 mm (Raypex 6, 0.0 mm, immature apex with divergent AF walls), respectively. Both Root ZX II and Raypex 6 exhibited mean errors comparable to those observed in previous studies when tested on fully formed complete apices. 15 16 EPex Pro and CanalPro had not been previously evaluated; nevertheless, their results aligned with those of previously tested devices.

The presence of large AFs did not significantly affect the accuracy of the electronic devices. This confirms findings from studies indicating good accuracy of EMs with matched files in teeth with incomplete/enlarged apices 17 18 19 or deciduous teeth. 20 21 Discrepancies in EFL accuracy may occasionally stem from a lack of apical fit of the instrument, especially in wide AFs, potentially interfering with capacitive impedance. However, in AFs with divergent canal walls, achieving apical fit becomes impossible, leading instruments to compensate for the reduction in capacitive factor. In this study, the tested EFLs exhibited similarity; however, Raypex 6 and CanalPro, utilizing similar mechanisms, displayed reduced accuracy with divergent AFs. This suggests that these electronic devices' mechanisms are impacted by compromised interpretation of the capacitive factor, resulting in a broader measurement error range.

Consequently, it can be inferred that EPex Pro and CanalPro offer a suitable accuracy akin to that provided by Root ZX II and Raypex 6. They stand as dependable tools for EMs even in nonideal foramen morphology scenarios. Concerning Raypex 6 and CanalPro interpretations, clinicians should consider their limitations when encountering anatomical variations like expansive apical foramen walls. Hence, endodontists must meticulously approach EMs in teeth with immature apices and divergent canal walls, where the use of EFLs might encounter limitations.

## Conclusion

Considering the study conditions, it was possible to conclude that the four EFLs tested, Root ZX II, Raypex 6, CanalPro, and EPex Pro, are reliable tools for determining the apical limit during endodontic treatment, regardless of the desired apical limit. However, unlike the others, Raypex 6 and CanalPro demonstrated an increase in precision related to foraminal morphology.
